# Template-Assisted Plasmonic Nanogap Shells for Highly Enhanced Detection of Cancer Biomarkers

**DOI:** 10.3390/ijms22041752

**Published:** 2021-02-10

**Authors:** Homan Kang, Sinyoung Jeong, Jin-Kyoung Yang, Ahla Jo, Hyunmi Lee, Eun Hae Heo, Dae Hong Jeong, Bong-Hyun Jun, Hyejin Chang, Yoon-Sik Lee

**Affiliations:** 1 Interdisciplinary Program in Nano-Science and Technology, Seoul National University, Seoul 08826, Korea; hkang7@mgh.harvard.edu (H.K.); jeongdh@snu.ac.kr (D.H.J.); 2 Department of Chemistry Education, Seoul National University, Seoul 08826, Korea; sinyoung.jeong0@gmail.com; 3 School of Chemical & Biological Engineering, Seoul National University, Seoul 08826, Korea; yjk0627@gmail.com (J.-K.Y.); 2hmlee2@gmail.com (H.L.); 4 Department of Bioscience and Biotechnology, Konkuk University, Seoul 05029, Korea; iamara0621@konkuk.ac.kr; 5 Division of Science Education, Kangwon National University, Chuncheon 24341, Korea; eunhae@kangwon.ac.kr

**Keywords:** surface-enhanced Raman scattering, nanogap shell, biomolecule detection, prostate-specific antigen, non-small cell lung cancer cell

## Abstract

We present a template-assisted method for synthesizing nanogap shell structures for biomolecular detections based on surface-enhanced Raman scattering. The interior nanogap-containing a silver shell structure, referred to as a silver nanogap shell (Ag NGS), was fabricated on silver nanoparticles (Ag NPs)-coated silica, by adsorbing small aromatic thiol molecules on the Ag NPs. The Ag NGSs showed a high enhancement factor and good signal uniformity, using 785-nm excitation. We performed in vitro immunoassays using a prostate-specific antigen as a model cancer biomarker with a detection limit of 2 pg/mL. To demonstrate the versatility of Ag NGS nanoprobes, extracellular duplex surface-enhanced Raman scattering (SERS) imaging was also performed to evaluate the co-expression of cancer biomarkers, human epidermal growth factor-2 (HER2) and epidermal growth factor receptor (EGFR), in a non-small cell lung cancer cell line (H522). Developing highly sensitive Ag NGS nanoprobes that enable multiplex biomolecular detection and imaging can open up new possibilities for point-of-care diagnostics and provide appropriate treatment options and prognosis.

## 1. Introduction

Surface-enhanced Raman scattering (SERS) has been applied to various analytical areas, especially in biosensing and bioimaging, due to its great advantages such as high sensitivity, multiplexing capability, and resistance to photobleaching [1,2,3,4,5,6,7]. Despite significant progress made with various types of SERS nanoprobes, intense investigations are still ongoing in terms of developing a practical use for SERS techniques. In previous years, various synthetic strategies have been developed for plasmonic nanogap structures for use as SERS substrates to improve signal sensitivity [8,9,10,11,12,13]. Intra nanogap structures, which have a nanometer-scale interior gap, are highly promising as SERS nanoprobes because they can exclude unwanted interference in media and because they have uniform and controllable nanogaps, resulting in strongly enhanced and tunable SERS signals [14,15,16,17,18,19]. To fabricate SERS-active interior nanogap structures, DNA-tethering methods [9,13,14,20,21], etching-assisted approaches [10,22], and dithiol molecular-anchoring methods [8,11,23] have been developed. However, challenges remain in using these approaches to control the positions of Raman labeling compounds in the plasmonic nanogaps and to control the size, shape, and functionalization of tethering molecules [17].

Quantitative detection and imaging of clinical tumor biomarkers are very important in aiding diagnosis, monitoring progression, and evaluating the efficacies of clinical interventions. For instance, serum prostate-specific antigen (PSA) is the most reliable and widely used clinical biomarker for diagnosing prostate cancer, and successful therapy for prostate cancer depends heavily on the detection of biomarkers [24,25]. In addition to detecting serum biomarkers for disease diagnosis, overexpressed cell-surface receptor tyrosine kinases, such as human epidermal growth factor-2 (HER2) and epidermal growth factor receptor (EGFR), have been used as predictive biomarkers for patients with different solid tumors, including non-small cell lung cancer (NSCLC). When HER2 and EGFR are overexpressed in patients with NSCLC, they exhibit aggressive tumor cell growth and enhanced sensitivity to EGFR tyrosine kinase inhibitors (TKIs). Thus, measuring EGFR and HER2 levels in patients with NSCLC has been evaluated to predict the anti-cancer activity of gefitinib (a TKI) due to increased HER2, in patients with EGFR-positive NSCLC. Previous findings suggest that detecting both receptors can improve disease prognosis for some patients by identifying those likely to benefit from an EGFR TKI [26,27,28].

Herein, we report a template-assisted method for synthesizing a silver nanogap shell (Ag NGS) structure containing a Raman label compound (RLC) layer in an interior nanogap. This structure was fabricated using a silver nanoparticles (NPs)-coated silica (Ag silica) core as a template. Owing to the assembled monolayer of aromatic thiol molecules, gap-generating pedestals, and RLCs on Ag silica surfaces, the interior nanogap layer could be generated by forming an additional Ag shell on top of the Ag silica template. Ag NGSs strongly enhanced SERS signals and were successfully applied for PSA detection and duplex-imaging of NSCLC cells overexpressing HER2.

## 2. Results and Discussion

### 2.1. Characterization of Interior Nanogap-Containing Ag NGSs

**Figure 1:**
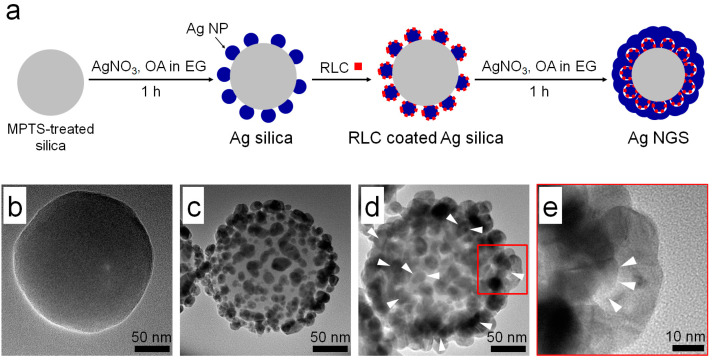
Synthesis and characterization of silver nanogap shell (Ag NGS) particles as near-infrared active surface-enhanced Raman scattering (SERS) nanoprobes. (**a**) Schematic illustration of the method used to prepare Ag NGS particles. Representative TEM images of (**b**) thiol-functionalized silica, (**c**) Ag silica, (**d**) Ag NGS, and (**e**) the interior gap structure of Ag NGS (white arrow heads) at high magnification.

Ag NGSs were synthesized as described in Figure 1a. First, the dielectric silica core template (150 nm in diameter) was prepared using the Stöber method and subsequently modified with 3-mercaptopropyl trimethoxysilane (MPTS) (Figure 1b). The resulting thiolated silica NP was decorated with silver nanoparticles (Ag NPs) (12.1 ± 2.9 nm in diameter) by performing in situ Ag ion reductions to obtain Ag NP-coated silica (Ag silica) (Figure 1c). Subsequently, a benzenethiol derivative 4-flourobenzenthiol (4-FBT) was introduced as an RLC on the Ag NP surface to form a self-assembled monolayer. To achieve interior nanogap structures, we adopted our previously developed method [29,30] for one-step Ag nanoshell (Ag NS) synthesis. The RLC-coated Ag silica was re-dispersed in ethylene glycol (EG). Then, AgNO_3_, octylamine (OA, a reducing agent), and poly(vinylpyrrolidone) (PVP; a surface-passivation agent) were added for additional Ag shell coating. As shown in Figure 1d, the RLC-coated Ag silica particles were successfully covered with an additional Ag shell on the surface. Ag silica has an absorption band typical of Ag NP at approximately 420 nm. In contrast, the extinction spectrum of Ag NGS (Figure S1) showed very broad absorption from 400 to 800 nm. This extinction property suggested that Ag NGS was suitable as a near-infrared (NIR) active SERS substrate and applicable for a wide range of laser photoexcitations. The shape and morphology of Ag NGSs did not differ significantly from those with gapless structures (Ag NSs), and the average size of the Ag NGSs was also similar to that of Ag NSs [30]. However, high-resolution transmission electron microscopy (TEM) images under high magnification revealed that they contained an interior nanogap structure (Figure 1e). Scanning electron microscopy (SEM) and dark-field microscopy confirmed that uniform Ag NGS particles were successfully fabricated (Figure S2).

### 2.2. Structural Analysis of the Time-and RLC-Dependent Formation of Ag NGSs

**Figure 2:**
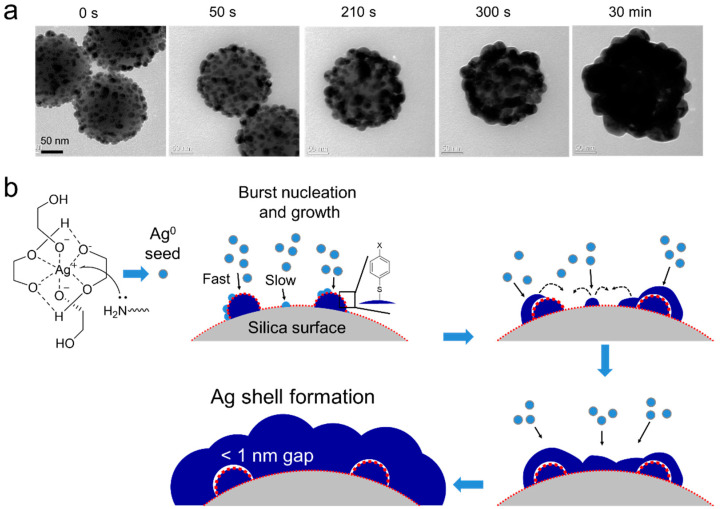
Observation and proposed mechanism of the shelling progress for Ag NGS structures. (**a**) TEM images of early-stage Ag NGS formation. Ag NPs coated on silica surfaces grew slowly at first, and additional seeding started after approximately 300 s. (**b**) Proposed mechanism of the Ag shelling progress starting from Ag silica. Ag^+^ ions in EG were easily reduced by octylamine using a chelating effect, and Ag^0^ rapidly induced the growth of Ag NPs on the silica surface. Ag^0^ approached the silica surface directly and slowly grew to form Ag NPs. Finally, the surface was fully covered with a robust Ag shell. Black line and dashed arrows indicate the nucleation and growth, respectively.

We used TEM to investigate the kinetics of Ag NGS formation and identify the process underlying the structural development (Figure 2a and Figure S3). Up to 60 s, it was difficult to observe any major changes, but after ~90 s, the morphology of the initial Ag silica structure started to change. Complete Ag shell-like structures appeared after 10 min and the reaction was terminated within 1 h. A detailed growth mechanism is proposed in Figure 2b. At first, the preformed Ag NPs grew slowly on the silica surface, and additional Ag NP seeds formed on the silica surface as time progressed. In terms of the growth of the Ag NPs, 4-FBT-coated Ag surfaces seemed to grow preferably over 4-FBT-coated silica surfaces. Finally, the newly formed Ag NPs and the Ag NPs grown on silica surfaces merged to form a fully covered shell structure. Although the overall rate of Ag shell formation was relatively slow compared to that of gapless Ag NSs (due to the presence of 4-FBTs on both Ag NP and the silica surface), the progress of Ag shell formation was similar to that of gapless Ag NSs [29,30]. These results suggest that we successfully applied our rapid Ag shell-formation method for nanogap-shell formation.

Additionally, we optimized conditions for maximizing the signal intensity by varying the 4-FBT concentration. Raman spectra of Ag silica solutions and Ag NGS solutions with different 4-FBT concentrations were measured at 532 nm and 785 nm, respectively, and were normalized to the signal intensity of the solvent (ethanol, 882 cm^−1^ band). The Raman signal intensities on the Ag silica were very slightly increased with 4-FBT concentrations above 2 mM (Figure S4), indicating that the Ag surface of Ag silica was saturated with 4-FBT at 2 mM. The Ag NGSs fabricated from the corresponding 4-FBT-labeled Ag silica particles exhibited the highest signal intensity, and their morphologies were nearly the same at higher concentrations. 

Next, we examined the effects of the structure of the RLC on Ag shell formation. Aromatic thiols are potential organic semiconductors, which allows close chemical contact with a metal substrate [31]. Differences in the degree of molecular inclination on the metal surface originating from variations in the intermolecular dipole–dipole orientation lead to different surface coverage of RLCs [32]. Therefore, changing the substituents of aromatic thiol RLCs could affect the template-assisted fabrication method used for generating Ag NGSs. First, we tested RLCs with a bulky substituent such as 4-isopropyl benzenethiol (4-IBT), which resulted in an incomplete shell formation and an irregular morphology (Figure S5). The long-chain molecule, methoxy poly(ethylene glycol) sulfhydryl, showed the same tendency. To examine the electronic effects of functional groups, we tested benzenethiols with electron-donating substituents (such as 4-aminophenol [4-ATP] and 3-mercaptobenzoic acid [3-MBA]) and electron-withdrawing substituents (such as 4-FBT, 4-chlorobenzenthiol [4-CBT], 4-bromobenzenthiol [4-BBT], and 2-chlorobenzenthiol [2-CBT]). The Ag shells were completely covered on the electron-withdrawing group containing RLC-coated Ag silicas, but not on the electron-donating group containing RLC-coated Ag silicas. Although the effects of substituents on Ag shell formation are not fully understood, we noticed that they could control Ag shell formation. When benzenethiol derivatives are adsorbed on the metal surface through metal–sulfur bonds, dipole interactions from various functional groups can affect Ag shelling processes. Measuring the SERS spectra of Ag NGSs labeled with BT, 2-CBT, 4-BBT, 4-CBT, and 2-FBT exhibited a capability for multiplex detection (Figure S6).

### 2.3. Characterization of SERS Sensitivities at the Single Particle Level and in Solution

**Figure 3:**
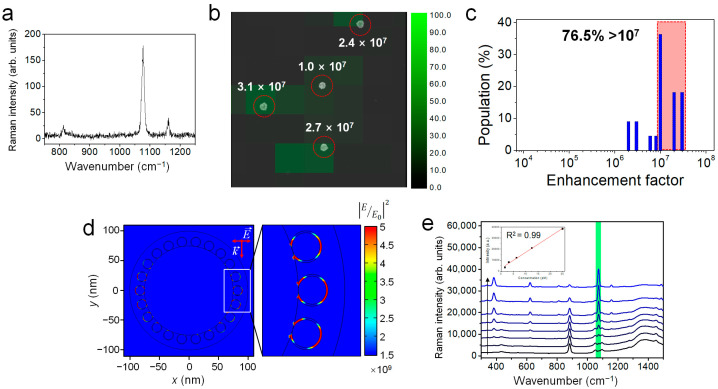
SERS analysis of Ag NGS particles at the single-particle level. (**a**) Typical SERS spectrum obtained from a single Ag NGS particle at an excitation wavelength of 785 nm. (**b**) SERS intensity map with enhancement factor (EF) values at 1075 cm^−1^ for 4-FBT. The intensity map was overlaid with the corresponding SEM image. The step size was 1 μm. (**c**) EF distribution of individual Au NGS particles (*n* = 22). The average EF value (*n* = 22) was 1.7 × 10^7^, and 76.5% of Ag NGS particles showed EF values over 10^7^. (**d**) Calculated near-field electromagnetic field distributions of Ag NGS using a 785-nm excitation laser. (**e**) Relationship between SERS intensities and Ag NGS concentrations in ethanol, as determined by serial dilution. For each spectrum, the Ag NGS concentration was 0.8, 1.6, 3.1, 6.3, 12.5, 25, 50, or 100 pM (starting from the bottom). The inset shows a linear relationship using Ag NGS samples at concentrations ranging from 1.6 to 25 pM.

The use of Ag NGS as a SERS probe was evaluated by single-particle SERS measurements, which were performed by point-by-point mapping on Ag NGS-dispersed glass slides. Then, SEM images were obtained and compared with the corresponding SERS-intensity maps to ensure that the SERS signals came from the individual Ag NGSs. A typical single SERS spectrum at an excitation wavelength of 785 nm is shown in Figure 3a, and the SERS intensity map for the 1075 cm^−1^ Raman band of the Ag NGS containing 4-FBT in the gap was overlaid with the corresponding SEM image (Figure 3b). The SERS enhancement factor (EF) for the Ag NGS can be calculated using the well-known equation, EF = (*I*_SERS_/*N*_SERS_)/(*I*_normal_/*N*_normal_), where *I*_SERS_ and *I*_normal_ are the intensities of a Raman band from SERS and normal Raman scattering, respectively, and *N*_normal_ and *N*_SERS_ are the numbers of 4-FBT in pure form and in self-assembled form on the Ag NGS surface, respectively. The Raman band at 1075 cm^−1^ for 4-FBT was used to estimate the EF. Ag NGS NPs dispersed on a patterned slide glass and neat 4-FBT (liquid) were used to measure *I*_SERS_ and *I*_normal_, respectively, using an identical laser power of ×100 objective lens (NA 0.90) for the EF calculation. To obtain *N*_normal_, a light collection volume of 18.8 μm^3^ was approximated from a cylindrical illuminated volume with a 2-μm diameter and a 6-μm height for normal Raman measurements. Using this approach, the SERS EF of 22 Ag NGS particles was calculated as 1.7 × 10^7^ on average. This value is quite high compared to representative EFs (1.5 × 10^5^–2.8 × 10^5^) of aromatic small molecules on Au nanorods that are commonly used as NIR SERS probes [33]. Remarkably, the EF values showed a narrow distribution, ranging from 1.1 × 10^7^ to 3.9 × 10^7^ for 76.5% of the measured population. All the analyzed particles displayed EFs of 2.1 × 10^6^ or more (Figure 3c), suggesting that Ag NGS enhanced the Raman signal with high sensitivity and uniformity. These findings suggest that Ag NGS contained an interior nanogap structure that was precisely controlled by a monolayer of aromatic thiol molecules. To support this result, we calculated the electric field (E-field) enhancements of the Ag NGS using the finite-element method (FEM) at λ = 785 nm (Figure 3d). The dimensions of the Ag NGS (Figure S7) were defined based on the TEM analysis shown in Figure 1. The calculation revealed that the E-field enhancement was highly maximized at the interior nanogaps (~1 nm) between the inner Ag NPs and the outer shell. Data from previous investigations performed to demonstrate gap-enhancement effects have suggested that electron transport (i.e., chemical enhancement) occurs for these three-dimensional intra-particle nanogaps, ranging in size from 0.7 to 2 nm, and that a non-quenching system could be achieved by controlling the tunneling effect between the nanogaps [34,35,36]. This FEM simulation was consistent with previous reports and successfully demonstrated why interior nanogap generation is critical for developing ultrasensitive SERS nanoprobes and why Ag NGS can generate strong SERS signals even from a single particle.

The detection sensitivity of Ag NGSs in the solution phase was measured by serial dilution. SERS signal intensities correlated with the concentrations of Ag NGSs (0.8, 1.6, 3.1, 6.3, 12.5, 25, 50, or 100 pM) in ethanol (Figure 3e). A linear relationship (R^2^ = 0.99) was obtained using the Ag NGS samples over the range of 1.6 to 25 pM, and the limit of detection was estimated to be approximately 1.6 pM. The photostability of Ag NGSs was also examined in the solution phase with 10 min of continuous laser exposure using 785-nm excitation (6.53 mW) (Figure S8). Raman spectra and signal intensities were consistent during the test and no significant changes of structural features were exhibited in the TEM images. Therefore, Ag NGSs were structurally and optically stable even under relatively strong laser power.

### 2.4. Immunoassay Using Magnetic Beads (MBs) for PSA Detection

As proof of concept, we demonstrated the feasibility of using Ag NGSs as highly sensitive optical nanoprobes for a PSA immunoassay. First, we coated the Ag NGS surface with silica to enhance the biocompatibility, stability, and solubility of Ag NGSs as efficient targeting nanoprobes. Silica does not interfere with the SERS signal of Ag NGSs due to its optical transparency. TEM images of prepared silica-coated Ag NGSs (Ag NGS@SiO_2_) are shown in Figure S9. Then, the Ag NGS@SiO_2_ nanoprobes were conjugated with a tracer anti-PSA antibody (TAb) using 1-ethyl-3-(3-dimethyl aminopropyl)-carbodiimide (EDC) [37]. To evaluate of bio-functionality of the TAb-Ag NGS@SiO_2_ nanoprobes, PSA-coated MBs were prepared and mixed with the TAb-Ag NGS@SiO_2_ solutions for 2 h. The mixture was subsequently washed by magnetic separation to remove the unbound TAb-Ag NGS@SiO_2_ nanoprobes (Figure S10a). Bovine serum albumin (BSA)-coated MBs were prepared as a negative control and were treated in the same manner. Subsequently, a 785-nm laser beam was focused on the MB pellets and SERS spectra were obtained. In the presence of PSA-coated MBs, strong SERS bands were observed at 1075 cm^−1^ that matched the band of Ag NGS itself (Figure S10b). In contrast, no signal was observed in the absence of PSA-coated MBs. These results show that the TAb-Ag NGS@SiO_2_ nanoprobes can be successfully applied for the immunological detection of PSA and that Ag NGS probes can selectively form immune complexes exclusively with PSA on the surface of MBs.

**Figure 4:**
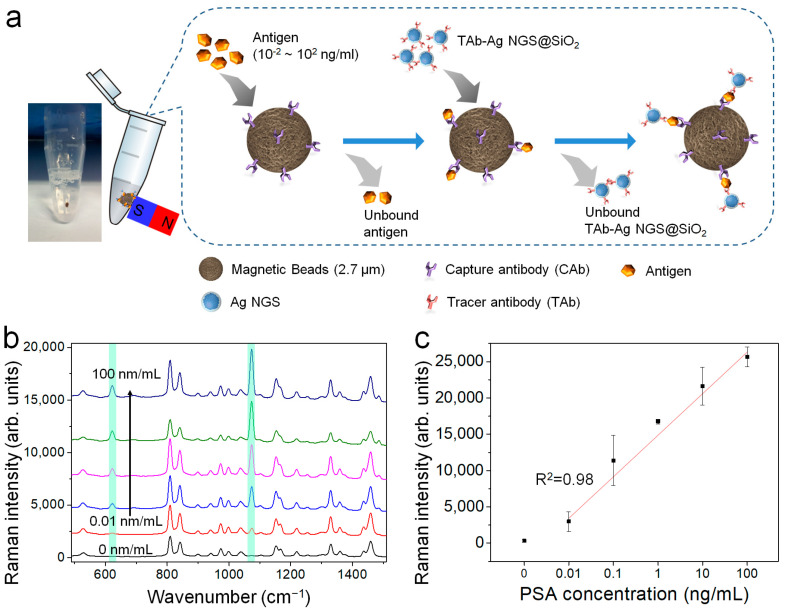
Quantitative detection of a prostate cancer biomarker (PSA) based on SERS spectroscopy, using a sandwich immunoassay method. (**a**) Schematic illustration of PSA detection using Ag NGS particles and magnetic beads (MBs). (**b**) SERS spectra of Ag NGS_[4-FBT]_@SiO_2_ particles conjugate to MBs, used to detect PSA at different concentrations (0 to 100 ng/mL). (**c**) Corresponding SERS intensities at 1075 cm^−1^ in (**b**) as a function of the logarithm of the PSA concentration.

Next, the sandwich immunoassay of PSA was performed with capture anti-PSA antibody (CAb)-conjugated MBs and TAb-Ag NGS_[4-FBT]_ by magnetic separation (Figure 4a). These immunoassays showed that the intensities of the SERS signals at 623 cm^−1^ and 1075 cm^−1^ (typical of 4-FBT) were strongly dependent on the PSA concentration (Figure 4b). At PSA concentrations ranging from 10^−2^ to 10^2^ ng/mL, the corresponding SERS intensities at 1075 cm^−1^ were obtained as a function of the logarithmic PSA concentration, as described by the following equation: *y* = 2412 log_10_(*x*) + 15682, where *x* = the PSA concentration in ng/mL (Figure 4c). The plot exhibited strong linearity (R^2^ = 0.98) over five orders of magnitude. The limit of detection (LOD) can be defined as an analyte concentration that produces a signal three-fold higher than that of the standard deviation of blank measurements (232 counts in this experiment). Therefore, the LOD was calculated as 2.0 pg/mL, which showed a much wider dynamic range with a lower LOD than recently reported for an enzyme-linked immunosorbent assay for PSA [38].

### 2.5. Cellular SERS Imaging of NSCLC Cells

**Figure 5:**
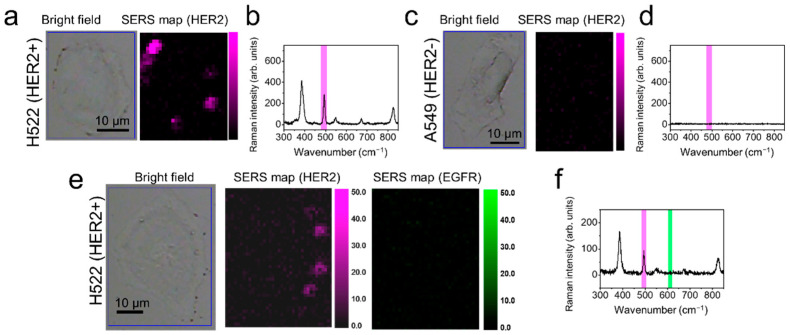
Extracellular human epidermal growth factor-2 (HER2)-specific detection of lung cancer cells (H522 and A549 cells). (**a**) H522 cells were treated with HER2-Ag NGSs_[2-FBT]_ particles. Bright-field image of H522 cells and a SERS intensity map corresponding to (**b**) a band at 494 cm^−1^ (HER2-Ag NGSs_[2-FBT]_, purple). (**c**) A549 cells treated with HER2-Ag NGSs_[2-FBT]_ were used as a negative control. Bright-field image of A549 cells and a SERS intensity map corresponding to (**d**) a band at 494 cm^−1^, obtained using an excitation wavelength of 785 nm, a laser power of 7.5 mW, and a light-acquisition time of 1 s. (**e**) H522 cells were treated with a mixture of HER2-Ag NGSs_[2-FBT]_ and epidermal growth factor receptor (EGFR)-Ag NGSs_[4-FBT]_ particles. Bright-field image of H522 cells and SERS intensity maps corresponding to (**f**) bands at 494 cm^−1^ (purple) and 623 cm^−1^ (green).

To expand the application of extracellular imaging and multiplex analysis, cell-based assays were performed using HER2-overexpressing H522 NSCLC cells and EGFR-overexpressing A549 cells (adenocarcinoma human alveolar basal epithelial cells) [39,40]. Previous data suggested that multiplex analysis of EGFR and HER2 expression could help in determining the prognosis of patients with NSCLC after gefitinib treatment [26,27,28]. Using the characteristic SERS signals of RLCs as a signature of specific biomarker proteins, SERS mapping for the expressed cancer biomarkers was performed using a confocal Raman microscope. To construct SERS maps, the SERS intensities at 623 cm^−1^ and 494 cm^−1^ (which correspond to 4-FBT and 2-FBT, respectively, and do not overlap) were used to distinguish both biomarkers. Figure 5 shows bright-field microscope images and SERS-mapping images (Figure 5a and Figure 5c, respectively), and the corresponding SERS spectra observed (Figure 5b and Figure 5d, respectively) in each cell treated with HER2-Ag NGSs_[2-FBT]_. As expected, HER2-positive H522 cells showed a clear 2-FBT signal (purple signal) after successful targeting with HER2-Ag NGSs_[2-FBT]_. The 2-FBT signal was not found with the HER2-negative A549 cell lines (used as a negative control). Figure S11a,c show the bright-field images and SERS maps of 4-FBT of EGFR-Ag NGS_[4-FBT]_-treated EGFR-positive A549 and EGFR-negative H522 cells (green signal). No 4-FBT signal was observed with H522 cells (Figure S11b), and only A549 cells showed a SERS signal at 623 cm^−1^ for 4-FBT (Figure S11d), revealing the specific targeting ability of EGFR-Ag NGS_[4-FBT]_. These results suggest that antibody-conjugated Ag NGS probes can selectively target biomarkers on cell surfaces. Consequently, H522 cells were treated with EGFR-Ag NGSs_[4-FBT]_ and HER2-Ag NGSs_[2-FBT]_ simultaneously. Only HER2-Ag NGSs_[2-FBT]_ targeted H522 cells, and Raman signals of 2-FBT were observed exclusively (Figure 5e,f). These results indicate that Ag NGSs could be used to validate biomarker expression and potentially as multiplex-capable SERS tags in bio-images with a strong signal.

## 3. Materials and Methods

### 3.1. Materials

Tetraethylorthosilicate (TEOS), 3-mercaptopropyl trimethoxysilane (MPTS), ethylene glycol (EG), poly(vinyl pyrrolidone) (PVP, Mw ~40,000), 1-ethyl-3-(3-dimethyl aminopropyl)-carbodiimide (EDC), *N*-hydroxy succinimide (NHS), *N*-methyl-2-pyrrolidone (NMP), *N*, *N*-diisopropylethylamine (DIPEA) silver nitrate (AgNO_3_, 99.99+%), octylamine (OA), ethanolamine, 2-fluorothiophenol (2-FBT), 4-fluorothiophenol (4-FBT), 4-aminobenzenthiol (4-ABT), 4-chlorobenzenethiol (4-CBT), 4-bromobenzenethiol (4-BBT), 2-(N-morpholino)ethanesulfonic acid (MES) buffer, phosphate-buffered saline (PBS), and bovine serum albumin (BSA) were purchased from Sigma-Aldrich (St. Louis, MO, USA) used without further purification. Methoxy poly(ethylene glycol) sulfhydryl (mPEG-SH) (Mw 5000) and carboxy-PEG-SH (Mw 5000) were purchased from Sun Bio (Anyang, Korea). NH_2_-PEG-COOH (Mw 600) was purchased from Nanocs Inc (New York, NY, USA). Ammonium hydroxide (NH_4_OH, 27%) and ethanol (98%) were purchased from Daejung (Busan, Korea). Anti-PSA1 antibody (capture antibody, 14801; 4B7), anti-PSA3 antibody (tracer antibody14803; 1B5), and goat anti-mouse IgG were obtained from Bore Da Biotech (Seongnam, South Korea). Anti-PSA scFv-hFc was kindly supplied by Junho Chung at Seoul National University Hospital. Magnetic beads (Dynabeads) were purchased from Invitrogen (Carlsbad, CA, USA). Deionized (DI) water was used for all the experiments.

### 3.2. Synthesis

#### 3.2.1. Preparation of Ag NGSs

MPTS-treated silica NPs were prepared as previously reported [29,30,41]. Before making the nanogap shell structure, Ag NPs were decorated onto the surface of MPTS-labeled silica using an in situ reduction method. One hundred milligrams of MPTS-treated silica NPs were dispersed in 100 mL of AgNO_3_ solution (3.5 mM in EG). An 82.7-μL aliquot of OA was then rapidly added to the dispersed MPTS-treated silica nanospheres. The resulting dispersion was stirred for 1 h at 25 °C. Then, the resulting Ag silica was washed five times by centrifugation and resuspension with ethanol. Subsequently, each RLC (2 mM in ethanol) was added to 1 mg of Ag silica, and after 1 h reaction, the resulting Raman labeled Ag silica particles were washed with ethanol. RLC-coated Ag silica was dispersed AgNO_3_ solution (16.6 mL) containing 5 mg of PVP in EG, after which OA was added. During the reaction, the concentrations of AgNO_3_, OA, and silica NPs were varied to identify optimal conditions for the complete formation of additional Ag shells. After this process, the optimal concentrations of AgNO_3_ (3.5 mM) and OA (5 mM) were identified and were used to further investigate the other variables important for Ag NGS formation.

#### 3.2.2. Silica Encapsulation of Ag NGSs and Antibody Conjugation for Immunoassays

Silica-coated Ag NGS (Ag NGS@SiO_2_) were prepared as reported previously [42]. Ag NGSs (1 mg) were dispersed in 1 mL of ethanol containing 0.15 mM 11-mercaptoundecanoic acid (11-MUA). The resulting mixture was shaken vigorously for 1 h at room temperature. The resulting 11-MUA-treated Ag NGSs were centrifuged and washed with ethanol. To encapsulate the Ag NGSs with a silica shell, Raman label-treated Ag NGSs (3 mg) were dispersed in 15 mL of dilute aqueous sodium silicate solution (0.036 *wt*% SiO_2_). The dispersion was stirred with a magnetic stirring bar for 15 h. Ethanol (60 mL) was added to the reaction mixture while mixing vigorously with a magnetic stirring bar, and the dispersion was stirred for an additional 3 h at room temperature to form a thin silica shell. Ammonium hydroxide (28–30%, 250 μL) and tetraethylorthosilicate (TEOS, 30 μL) were added to the above mixture and stirred for an additional 24 h. The resulting Ag NGS@SiO_2_ particles were centrifuged, washed several times with ethanol, and dispersed in 3 mL ethanol.

To bio-functionalize the Ag NGS@SiO_2_ particles, the following surface-modification steps were performed. Ag NGS@SiO_2_ (1 mg) was dispersed in (3-aminopropyl) triethoxysilane (APTES) in ethanol (1 mL, 1 *v/v*%), followed by the addition of 10 μL NH_4_OH (27%). The resulting mixture was shaken for 1 h, washed several times with ethanol, and then redispersed in 500 μL *N*-methyl-2-pyrrolidone (NMP). Succinic anhydride (1.75 mg) was added to the dispersion of APTES-treated Ag NGS@SiO_2_ particles, after which 3.05 μL of *N*, *N*-diisopropylethylamine (DIPEA) was added to introduce a carboxyl group. The resulting mixture was stirred for 2 h at room temperature. Subsequently, the carboxyl group-functionalized Ag NGS@SiO_2_ particles were washed with deionized (DI) water and redispersed in 700 μL of 500 mM MES (pH 7). To activate the carboxyl group, sulfo-*N*-hydroxy succinimide (NHS) (2 mg) and EDC (2 mg) were added to the dispersion. The resulting dispersion was stirred for 30 min at room temperature and then washed with 50 mM 2-(N-morpholino) ethanesulfonic acid (MES). Activated Ag NGS@SiO_2_ was dispersed in 1 mL 50 mM MES, and then NH_2_-PEG-COOH (10 μL) was added to the dispersion. After a 2-h reaction with shaking, the resulting mixture was centrifuged and washed with 50 mM MES, and the unreacted carboxyl groups were blocked with ethanolamine (30 min). The surface-modified Ag NGS@SiO_2_ particles were washed with DI water and then redispersed in 700 μL of 500 mM MES (pH 7). Sulfo-NHS (2 mg) and EDC (2 mg) were added to the dispersion, and the resulting dispersion was stirred at room temperature for 30 min and then washed with 50 mM MES. TAb (150 μg) was added to the NHS-activated Ag NGS@SiO_2_ dispersion. The mixture was incubated for 2 h at room temperature. The resulting dispersion was washed with 50 mM MES, and the unreacted carboxyl groups were blocked with ethanolamine (30 min). The TAb-conjugated Ag NGS@SiO_2_ (TAb-Ag NGS@SiO_2_) particles were redispersed in phosphate-buffered saline (PBS) after incubation in PBS containing 5% bovine serum albumin (BSA; 5 *w/w*%). After removing the excess reagents by centrifugation and washing, TAb-Ag NGS@SiO_2_ was dispersed in PBS and stored at 4 °C before use.

To evaluate TAb-Ag NGS@SiO_2_ as an antigen-specific targeting nanoprobe, antigen-conjugated MBs were prepared. The epoxy-functionalized MB (2.85 μm diameter) was mixed overnight with CAb and vacant sites were blocked by adding BSA. BSA-conjugated MBs were prepared in the same manner as a negative control.

#### 3.2.3. Surface Modification of Ag NGSs for Extracellular Biomarker Detection

To enhance the water compatibility and functionality of Ag NGSs, the Ag NGSs surface was modified with carboxyl methoxy poly (ethylene glycol) sulfhydryl (PEG-SH). One milliliter carboxyl PEG-SH (2 mM in ethanol) was mixed with 1 mg Ag NGS for 1 h, followed by several cycles of centrifugation and resuspension in PBS (pH 7.4). COOH-PEGylated Ag NGSs were activated with 2 mM EDC and 5 mM NHS in 0.1 M PBS (pH 6.0). Then, 20 μg antibodies against EGFR and HER2 were added to the dispersion of activated PEGylated Ag NGSs. The mixture was shaken for 2 h, and antibody-conjugated Ag NGSs were washed several times with 0.1 M of PBS (pH 7.0) and PBS containing 0.1 *wt*% Tween-20 (TPBS).

### 3.3. Theoretical Calculation of the E-field Enhancement of Ag NGS

COMSOL Multiphysics software (Burlington, MA, USA) was used for the FEM calculations. The model dimensions used for the Ag NGS’s silica backbone diameter (150 nm), interior Ag NP diameter (15 nm), interior nanogap (1 nm), and thickness or outer silver shell (25 nm) were based on our TEM analysis (Figure 1). The incident radiation was along the *y*-axis, with polarization along the *x*-axis. The E-field distribution was calculated as |*E*/*E*_0_|^2^, where *E* is the magnitude of the scattered electromagnetic field and *E*_0_ is the magnitude of the incident electromagnetic field. The target structure was excited at λ = 785 nm with 1 mW.

### 3.4. SERS Measurements and Single-Particle Identification

For SERS single-particle measurements, Ag NGS suspensions (0.1 mg/mL in ethanol) were dropped on a patterned glass slide. A SERS intensity map was obtained by point-by-point mapping with a 1 μm step size, using a confocal micro-Raman system (LabRam 300) from JY-Horiba (Longjumeau, France) equipped with a BX41 optical microscope (Olympus). Raman scattering signals were collected in a back-scattering geometry using a ×100 objective lens (NA 0.90) and detected using a spectrometer equipped with a thermo-electrically cooled (−70 °C) charge-coupled device detector. Each spectrum in the SERS intensity map was obtained with a 785-nm photo-excitation laser power of 28 μW and a 10-s acquisition time. After each SERS measurement, the same field of view in the SERS intensity map was observed using a JSM-6701F field emission-scanning electron microscope (JEOL Ltd., Tokyo, Japan) to ensure that the SERS signals were generated from single particles. For SERS measurements in PSA immunoassays, an optical fiber-coupled portable Raman system (i-Raman) from B&W TEK (Newark, DE, USA) was used with an excitation wavelength of 785 nm, a laser power of 90 mW, and an acquisition time of 30 s. With the portable Raman system, the diffraction grating limited the spectral range to 175–3200 cm^−1^ with a spectral resolution of 3 cm^−1^. The maximal output power of the diode laser at the source was 300 mW. For photostability test of Ag NGSs, a confocal micro-Raman system (LabRam HR) from JY-Horiba was used in Kangwon Radiation Convergence Research Support Center of Korea Basic Science Institute (KBSI) at Kangwon National University.

### 3.5. Sandwich PSA Immunoassay Using Ag NGS Nanoprobes

Twenty-five microliters of Cab-conjugated MBs (Cab-MBs, 8 × 10^6^ /mL) were dispersed in 425 μL of 1% BSA/PBS solution (0.1 M, pH 7.0). In addition, PSA solutions at different concentrations (10‒100,000 pg/mL) were added to the Cab-MB such that their final concentrations ranged from 1 to 10,000 pg/mL. The resulting mixtures were incubated for 2 h under gentle shaking at room temperature. After washing the PSA-conjugated Cab-MBs several times with 0.1% PBS-T and PBS, the PSA–MB complexes were dispersed in 480 μL 1% BSA/PBS, followed by incubation with TAb-Ag NGS@SiO_2_ nanoprobes (10 μL, 0.4 mg/mL) for 2 h. Each mixture was washed several times with 0.1% PBS-T (0.1 M, pH 7.4) and PBS (0.1 M, pH 7.4) to remove the unbound TAb-Ag NGS@SiO_2_ nanoprobes. Next, a 785-nm laser beam was focused on the MB pellets and SERS spectra were obtained.

### 3.6. Cell-Based Assay Using Ag NGS Nanoprobes

The A549 (adenocarcinoma human alveolar basal epithelial) and H522 (human NSCLC) cell lines were obtained from the Korean Cell Line Bank (Seoul, South Korea). A549 cells were grown in F-12 complete medium and H522 cells were grown in RPMI-1640 complete medium. The media were supplemented with 10% fetal bovine serum and 1% penicillin/streptomycin (Gibco BRL, Grand Island, NY, USA). The cells were seeded into each well of Labteck 8-well slide chamber at a density of 20,000 cells in 300 μL media. The cells were incubated at 37 °C in a humidified atmosphere containing 5% CO_2_. After 24 h, the cells were washed with sterile PBS (pH 7.4) and fixed with 4% paraformaldehyde for 15 min. Then, the cells were washed with sterile PBS (pH 7.4) and incubated with 5% BSA for 1 h.

The surfaces of the Ag NGSs_[4-FBT]_ and Ag NGSs_[2-FBT]_ particles were functionalized with carboxylate groups using carboxy-PEG-thiol. Antibodies against HER2 and EGFR were conjugated to Ag NGSs_[2-FBT]_ and Ag NGSs_[4-FBT]_ particles using the EDC/NHS method, respectively. Next, HER2-Ag NGSs_[2-FBT]_ (15 µg) and EGFR-Ag NGSs_[4-FBT]_ (15 µg) particles were incubated with A549 and H522 cells for 2 h. Then, the cells were rinsed several times with 0.1% TPBS, PBS (pH 7.4), and DI water.

## 4. Conclusions

In summary, we developed a novel approach for fabricating a highly sensitive and reliable SERS nanoprobe, Ag NGS, which has an interior nano-gap structure. A monolayer of aromatic thiol molecules as RLCs on Ag silica surfaces enabled us to generate nanogap structures when introducing additional Ag shells, which can produce strong Raman signals at the single-particle level. Ag NGS nanoprobes showed a high enhancement factor (1.7 × 10^7^ on average) and a signal uniformity at the 785-nm excitation wavelength, owing to the nanogaps of Ag NGSs. Ag NGS was successfully applied in an immunoassay for PSA detection with an LOD of 2 pg/mL. In addition, multiplexed cellular SERS images were successfully obtained with the H522 NSCLC cell line. Our method for fabricating Ag NGS is also applicable to various RLCs, enabling multiplex detection of a serum biomarker (PSA) and validation of the cellular expression of biomarkers (EGFR and HER2).

## Data Availability

Not applicable.

## Supplementary Materials

The following are available online at https://www.mdpi.com/1422-0067/22/4/1752/s1, Figure S1: UV/Vis/NIR extinction spectra of Ag nanogap-shells and Ag silica, Figure S2: (a) FE-SEM images and (b) dark-filed microscopic image of Ag NGSs, Figure S3: TEM images of Ag NGS particles taken during the Ag shelling process to investigate the mechanism and kinetics of nanogap shell formation, Figure S4: SERS spectra and normalized SERS signal intensities of Ag silica and Ag NGS particles after 4-FBT labeling, Figure S5: TEM images of various Raman label compound (RLC)-labeled Ag NGSs showing the formation and surface morphology of Ag NGSs, Figure S6: SERS spectra of Ag NGSs labeled with BT, 2-CBT, 4-BBT, 4-CBT, or 2-FBT, Figure S7: Dimensions of Ag NGS particles, as defined for E-field enhancement calculations, Figure S8: TEM images of silica-encapsulated Ag NGSs, Figure S9: Conjugating PSA to epoxy-functionalized magnetic beads (MBs) to validate the Tab-Ag NGS@SiO_2_ probes, Figure S10: Bright-field images and corresponding SERS-mapping images for two different types of lung cancer cell lines.

## References

[1] Zong C., Xu M., Xu L.J., Wei T., Ma X., Zheng X.S., Hu R., Ren B. (2018). Surface-Enhanced Raman Spectroscopy for Bioanalysis: Reliability and Challenges. Chem. Rev..

[2] Pallaoro A., Braun G.B., Moskovits M. (2015). Biotags Based on Surface-Enhanced Raman Can Be as Bright as Fluorescence Tags. Nano Lett..

[3] Cheng Z., Choi N., Wang R., Lee S., Moon K.C., Yoon S.Y., Chen L., Choo J. (2017). Simultaneous Detection of Dual Prostate Specific Antigens Using Surface-Enhanced Raman Scattering-Based Immunoassay for Accurate Diagnosis of Prostate Cancer. ACS Nano.

[4] Chang H., Kang H., Yang J.K., Jo A., Lee H.Y., Lee Y.S., Jeong D.H. (2014). Ag shell-Au satellite hetero-nanostructure for ultra-sensitive, reproducible, and homogeneous NIR SERS activity. ACS Appl. Mater. Interfaces.

[5] Kang H., Koh Y., Jeong S., Jeong C., Cha M.G., Oh M., Yang J.-K., Lee H., Jeong D.H., Jun B.-H. (2020). Graphical and SERS Dual-Modal Identifier for Encoding OBOC Library. Sens. Actuators B Chem..

[6] Koo K.M., Wang J., Richards R.S., Farrell A., Yaxley J.W., Samaratunga H., Teloken P.E., Roberts M.J., Coughlin G.D., Lavin M.F. (2018). Design and Clinical Verification of Surface-Enhanced Raman Spectroscopy Diagnostic Technology for Individual Cancer Risk Prediction. ACS Nano.

[7] Wang J., Koo K.M., Wang Y., Trau M. (2019). Engineering State-of-the-Art Plasmonic Nanomaterials for SERS-Based Clinical Liquid Biopsy Applications. Adv. Sci..

[8] Yang T., Jiang J. (2016). Embedding Raman Tags between Au Nanostar@Nanoshell for Multiplex Immunosensing. Small.

[9] Kang J.W., So P.T., Dasari R.R., Lim D.K. (2015). High resolution live cell Raman imaging using subcellular organelle-targeting SERS-sensitive gold nanoparticles with highly narrow intra-nanogap. Nano Lett..

[10] Jaiswal A., Tian L., Tadepalli S., Liu K.K., Fei M., Farrell M.E., Pellegrino P.M., Singamaneni S. (2014). Plasmonic nanorattles with intrinsic electromagnetic hot-spots for surface enhanced Raman scattering. Small.

[11] Gandra N., Singamaneni S. (2013). Bilayered Raman-intense gold nanostructures with hidden tags (BRIGHTs) for high-resolution bioimaging. Adv. Mater..

[12] Sun L., Sung K.-B., Dentinger C., Lutz B., Nguyen L., Zhang J., Qin H., Yamakawa M., Cao M., Lu Y. (2007). Composite organic-inorganic nanoparticles as Raman labels for tissue analysis. Nano Lett..

[13] Lim D.K., Jeon K.S., Hwang J.H., Kim H., Kwon S., Suh Y.D., Nam J.M. (2011). Highly uniform and reproducible surface-enhanced Raman scattering from DNA-tailorable nanoparticles with 1-nm interior gap. Nat. Nanotech..

[14] Oh J.W., Lim D.K., Kim G.H., Suh Y.D., Nam J.M. (2014). Thiolated DNA-based chemistry and control in the structure and optical properties of plasmonic nanoparticles with ultrasmall interior nanogap. J. Am. Chem. Soc..

[15] Bao Z., Zhang Y., Tan Z., Yin X., Di W., Ye J. (2018). Gap-enhanced Raman tags for high-contrast sentinel lymph node imaging. Biomaterials.

[16] Zhang Y., Qiu Y., Lin L., Gu H., Xiao Z., Ye J. (2017). Ultraphotostable Mesoporous Silica-Coated Gap-Enhanced Raman Tags (GERTs) for High-Speed Bioimaging. ACS Appl. Mater. Interfaces.

[17] Nam J.M., Oh J.W., Lee H., Suh Y.D. (2016). Plasmonic Nanogap-Enhanced Raman Scattering with Nanoparticles. Acc. Chem. Res..

[18] Hu C., Shen J., Yan J., Zhong J., Qin W., Liu R., Aldalbahi A., Zuo X., Song S., Fan C. (2016). Highly narrow nanogap-containing Au@Au core-shell SERS nanoparticles: Size-dependent Raman enhancement and applications in cancer cell imaging. Nanoscale.

[19] Zhang Y., Gu Y., He J., Thackray B.D., Ye J. (2019). Ultrabright gap-enhanced Raman tags for high-speed bioimaging. Nat. Commun..

[20] Zhao B., Shen J., Chen S., Wang D., Li F., Mathur S., Song S., Fan C. (2014). Gold nanostructures encoded by non-fluorescent small molecules in polyA-mediated nanogaps as universal SERS nanotags for recognizing various bioactive molecules. Chem. Sci..

[21] Shen J., Su J., Yan J., Zhao B., Wang D., Wang S., Li K., Liu M., He Y., Mathur S. (2014). Bimetallic nano-mushrooms with DNA-mediated interior nanogaps for high-efficiency SERS signal amplification. Nano Res..

[22] Kim M., Ko S.M., Kim J.M., Son J., Lee C., Rhim W.K., Nam J.M. (2018). Dealloyed Intra-Nanogap Particles with Highly Robust, Quantifiable Surface-Enhanced Raman Scattering Signals for Biosensing and Bioimaging Applications. ACS Cent. Sci..

[23] Lin L., Gu H., Ye J. (2015). Plasmonic multi-shell nanomatryoshka particles as highly tunable SERS tags with built-in reporters. Chem. Commun..

[24] Madu C.O., Lu Y. (2010). Novel diagnostic biomarkers for prostate cancer. J. Cancer.

[25] Clarke R.A., Schirra H.J., Catto J.W., Lavin M.F., Gardiner R.A. (2010). Markers for detection of prostate cancer. Cancers.

[26] Cappuzzo F., Varella-Garcia M., Shigematsu H., Domenichini I., Bartolini S., Ceresoli G.L., Rossi E., Ludovini V., Gregorc V., Toschi L. (2005). Increased HER2 gene copy number is associated with response to gefitinib therapy in epidermal growth factor receptor–positive non–small-cell lung cancer patients. J. Clin. Oncol..

[27] Daniele L., Macrì L., Schena M., Dongiovanni D., Bonello L., Armando E., Ciuffreda L., Bertetto O., Bussolati G., Sapino A. (2007). Predicting gefitinib responsiveness in lung cancer by fluorescence in situ hybridization/chromogenic in situ hybridization analysis of EGFR and HER2 in biopsy and cytology specimens. Mol. Cancer Ther..

[28] Hirsch F., Varella-Garcia M., Cappuzzo F. (2009). Predictive value of EGFR and HER2 overexpression in advanced non-small-cell lung cancer. Oncogene.

[29] Yang J.-K., Kang H., Lee H., Jo A., Jeong S., Jeon S.-J., Kim H.-I., Lee H.-Y., Jeong D.H., Kim J.-H. (2014). Single-Step and Rapid Growth of Silver Nanoshells as SERS-Active Nanostructures for Label-Free Detection of Pesticides. ACS Appl. Mater. Interfaces.

[30] Kang H., Yang J.-K., Noh M.S., Jo A., Jeong S., Lee M., Lee S., Chang H., Lee H., Jeon S.-J. (2014). One-Step Synthesis of Silver Nanoshell with Bumps for Highly Sensitive Near-IR SERS Nanoprobes. J. Mater. Chem. B.

[31] Hong S.Y., Yeh P.C., Dadap J.I., Osgood R.M. Jr. (2012). Interfacial dipole formation and surface-electron confinement in low-coverage self-assembled thiol layers: thiophenol and p-fluorothiophenol on Cu(111). ACS Nano.

[32] Jiang P., Deng K., Fichou D., Xie S.S., Nion A., Wang C. (2009). STM imaging ortho- and para-fluorothiophenol self-assembled monolayers on Au(111). Langmuir.

[33] Jin X., Khlebtsov B.N., Khanadeev V.A., Khlebtsov N.G., Ye J. (2017). Rational Design of Ultrabright SERS Probes with Embedded Reporters for Bioimaging and Photothermal Therapy. ACS Appl. Mater. Interfaces.

[34] Scholl J.A., García-Etxarri A., Koh A.L., Dionne J.A. (2013). Observation of Quantum Tunneling between Two Plasmonic Nanoparticles. Nano Lett..

[35] Lin L., Zapata M., Xiong M., Liu Z., Wang S., Xu H., Borisov A.G., Gu H., Nordlander P., Aizpurua J. (2015). Nanooptics of Plasmonic Nanomatryoshkas: Shrinking the Size of a Core–Shell Junction to Subnanometer. Nano Lett..

[36] Yang L., Wang H., Fang Y., Li Z. (2016). Polarization State of Light Scattered from Quantum Plasmonic Dimer Antennas. ACS Nano.

[37] Chang H., Kang H., Ko E., Jun B.-H., Lee H.-Y., Lee Y.-S., Jeong D.H. (2016). PSA Detection with Femtomolar Sensitivity and a Broad Dynamic Range Using SERS Nanoprobes and an Area-Scanning Method. ACS Sens..

[38] Gao Z., Hou L., Xu M., Tang D. (2014). Enhanced colorimetric immunoassay accompanying with enzyme cascade amplification strategy for ultrasensitive detection of low-abundance protein. Sci. Rep..

[39] Menju T., Hashimoto S., Hashimoto A., Otsuka Y., Handa H., Ogawa E., Toda Y., Wada H., Date H., Sabe H. (2011). Engagement of overexpressed Her2 with GEP100 induces autonomous invasive activities and provides a biomarker for metastases of lung adenocarcinoma. PLoS ONE.

[40] Ono M., Hirata A., Kometani T., Miyagawa M., Ueda S., Kinoshita H., Fujii T., Kuwano M. (2004). Sensitivity to gefitinib (Iressa, ZD1839) in non-small cell lung cancer cell lines correlates with dependence on the epidermal growth factor (EGF) receptor/extracellular signal-regulated kinase 1/2 and EGF receptor/Akt pathway for proliferation. Mol. Cancer Ther..

[41] Kang H., Jeong S., Park Y., Yim J., Jun B.-H., Kyeong S., Yang J.-K., Kim G., Hong S., Lee L.P. (2013). Near-infrared SERS nanoprobes with plasmonic Au/Ag hollow-shell assemblies for in vivo multiplex detection. Adv. Funct. Mater..

[42] Mir-Simon B., Reche-Perez I., Guerrini L., Pazos-Perez N., Alvarez-Puebla R.A. (2015). Universal One-Pot and Scalable Synthesis of SERS Encoded Nanoparticles. Chem. Mater..

