# Supplementary material for: Template-Assisted Plasmonic Nanogap Shells for Highly Enhanced Detection of Cancer Biomarkers
Source: Int J Mol Sci. 2021 Feb 10;22(4):1752. doi: 10.3390/ijms22041752 (PMC7916425; doi:10.3390/ijms22041752)
Supplement: Supplementary file 1 [file ijms-22-01752-s001.pdf]

Supplementary Material

Template-Assisted Plasmonic Nanogap-Shells for Highly Enhanced Biomarker Detection

Homan Kang, Sinyoung Jeong, Jin-Kyoung Yang, Ahla Jo, Hyunmi Lee, Eun Hae Heo, Dae Hong Jeong, Bong-Hyun Jun, Hyejin Chang,* and Yoon-Sik Lee**

***Corresponding Authors:** B-H.J. (bjun@konkuk.ac.kr), H.C. (hjchang@kangwon.ac.kr) or Y-S.L. (yslee@snu.ac.kr)

List of Contents

1. Figure S1. UV/Vis/NIR extinction spectra of Ag nanogap-shells and Ag silica
2. Figure S2. (a) FE-SEM images and (b) dark-filed microscopic image of Ag NGSs
3. Figure S3. TEM images of Ag NGS particles taken during the Ag shelling pro cess to investigate the mechanism and kinetics of nanogap shell formation
4. Figure S4. SERS spectra and normalized SERS signal intensities of Ag silica a nd Ag NGS particles after 4-FBT labeling
5. Figure S5. TEM images of various Raman label compound (RLC)-labeled Ag NGSs showing the formation and surface morphology of Ag NGSs
6. Figure S6. SERS spectra of Ag NGSs labeled with BT, 2-CBT, 4-BBT, 4-CBT, or 2-FBT
7. Figure S7. Dimensions of Ag NGS particles, as defined for E-field enhanceme nt calculations
8. Figure S8. Photostability of Ag NGS particles.
9. Figure S9. TEM images of silica-encapsulated Ag NGSs
10. Figure S10. Conjugating PSA to epoxy-functionalized magnetic beads (MBs) to validate the Tab-AgNGS@SiO2 probes
11. Figure S11. Bright-field images and corresponding SERS-mapping images for t wo different types of lung cancer cell lines

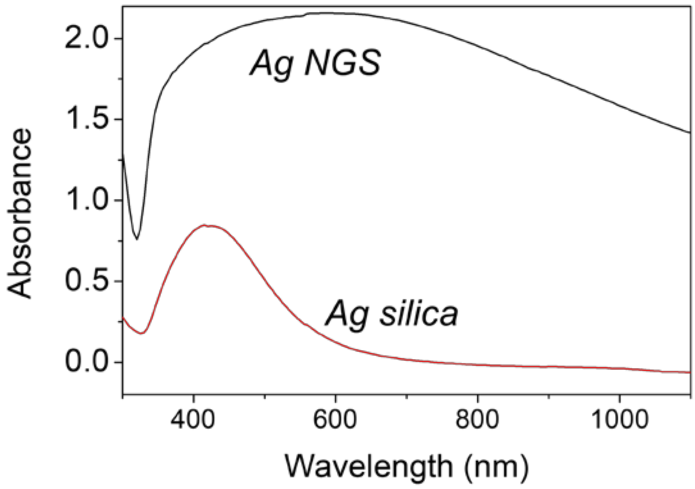

Figure S1. Ultraviolet/visible/NIR extinction spectra of Ag nanogap shells (black line) and Ag silica (red line) at the same concentration (0.06 mg/mL, based on silica NPs). The extinction coefficients of Ag NGS and Ag silica at 785 nm were obtained to 4.55×10^{10} cm^{-1} M^{-1} and 0 cm^{-1} M^{-1}, respectively.

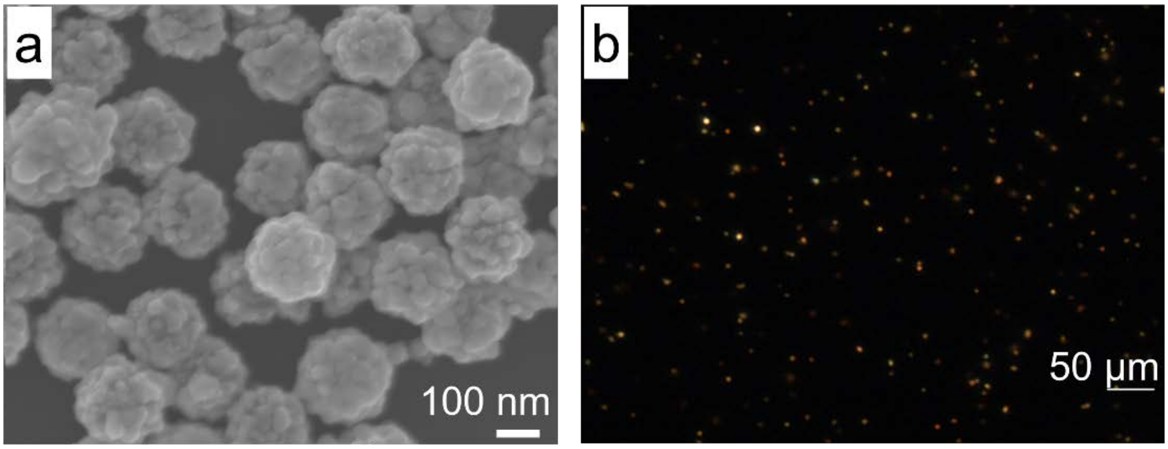

Figure S2. (a) Field emission-SEM image of Ag NGSs and (b) dark-filed microscopic image of dispersed Ag NGSs on a glass slide

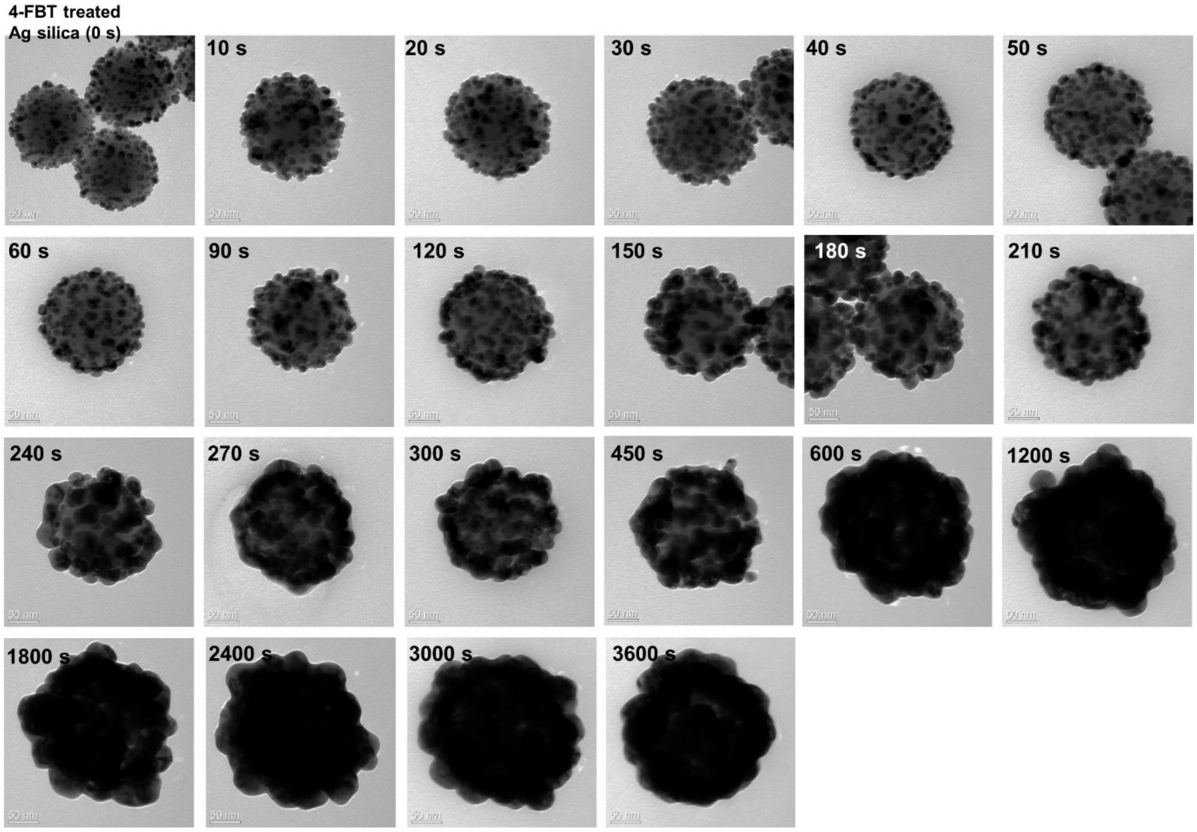

Figure S3. TEM images of Ag NGS particles taken during the Ag shelling process to investigate the mechanism and kinetics of nanogap shell formation

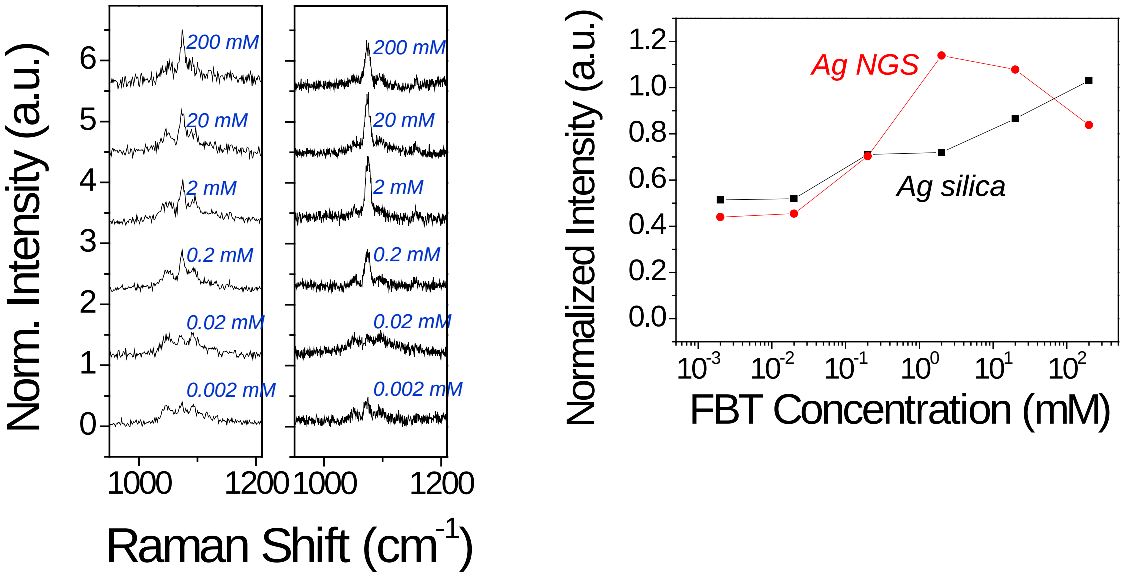

Figure S4. SERS spectra and normalized SERS signal intensities of Ag silica and Ag NGS particles after 4-FBT labeling. (a) SERS spectra of Ag silica and Ag NGS particles labeled in solution with various concentrations of 4-FBT (0.002–200 mM). SERS spectra were obtained using a 532-nm laser with 1 mW power (for Ag silica) or a 785-nm laser with 5.4 mW power (for Ag NGSs). The laser line was selected for the corresponding localized surface plasmon resonance of each type of particles. (b) Normalized SERS signal intensities of Ag silica and Ag NGS particles at 1075 cm^{-1}. Each signal was normalized by dividing by the ethanol peak intensity at 886 cm^{-1}.

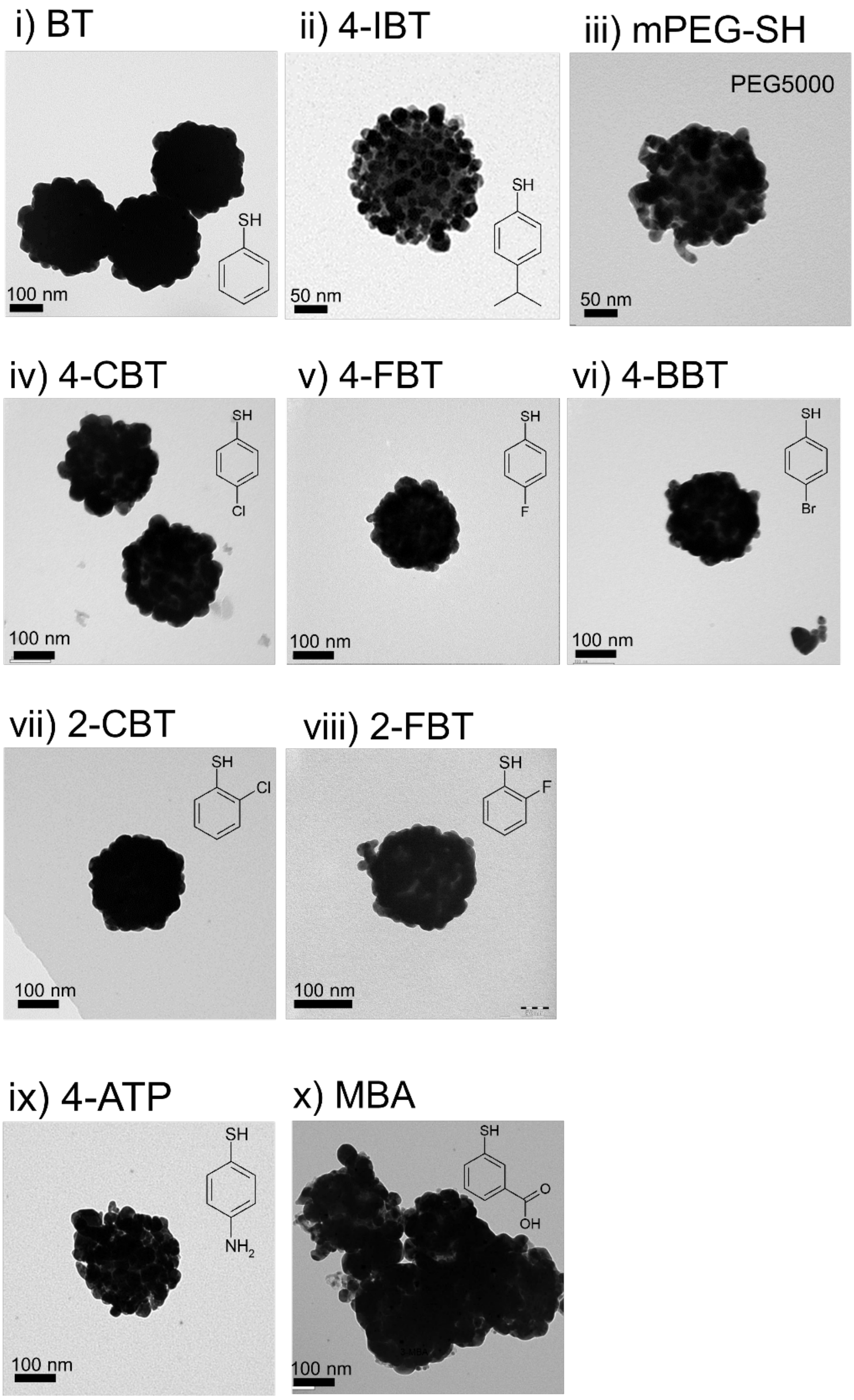

Figure S5. TEM images of various Raman label compound (RLC)-labeled Ag NGSs showing the formation and surface morphology of Ag NGSs. TEM images were taken to investigate the effects of different RLCs.

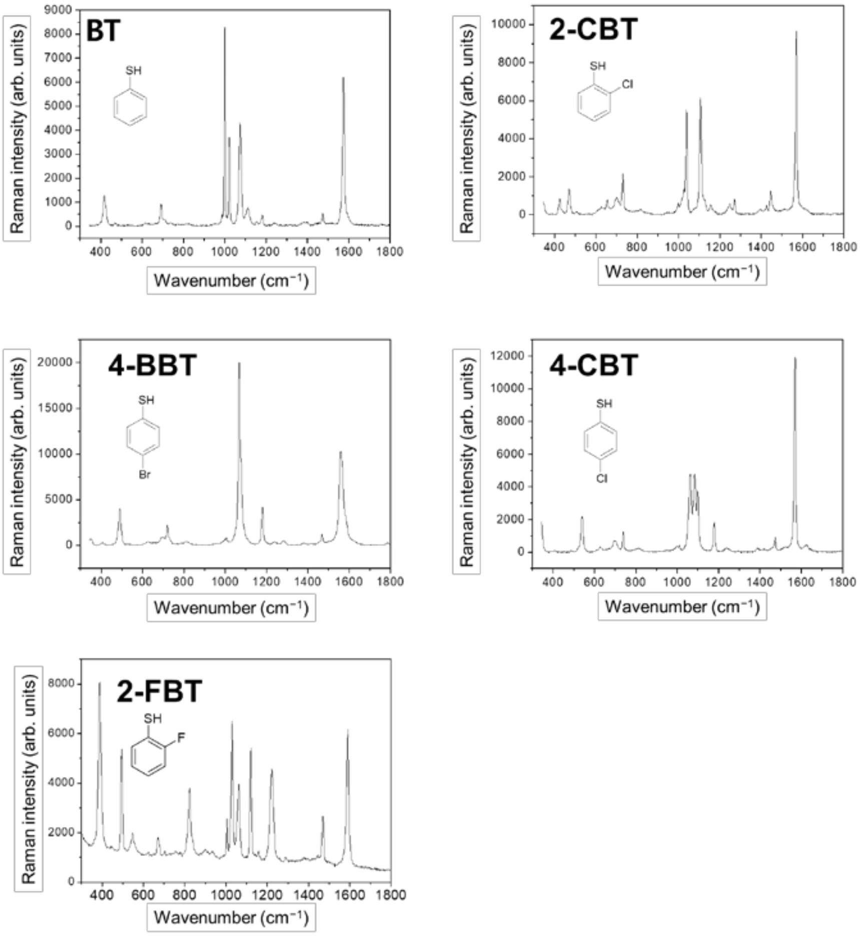

Figure S6. SERS spectra of BT, 2-CBT, 4-BBT, 4-CBT, and 2-FBT labeled Ag NGSs.

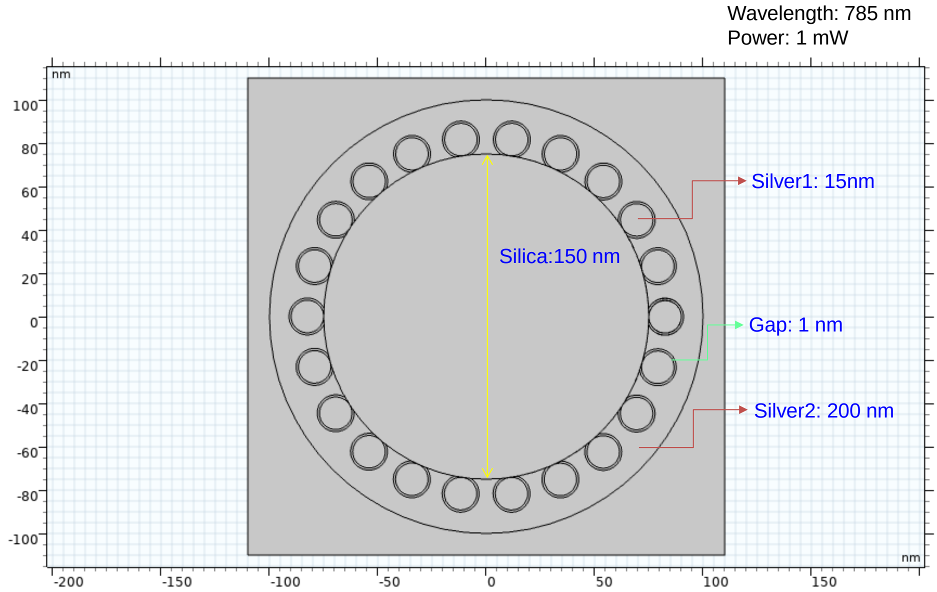

Figure S7. Dimensions of Ag NGS particles, as defined for the E-field enhancement calculations

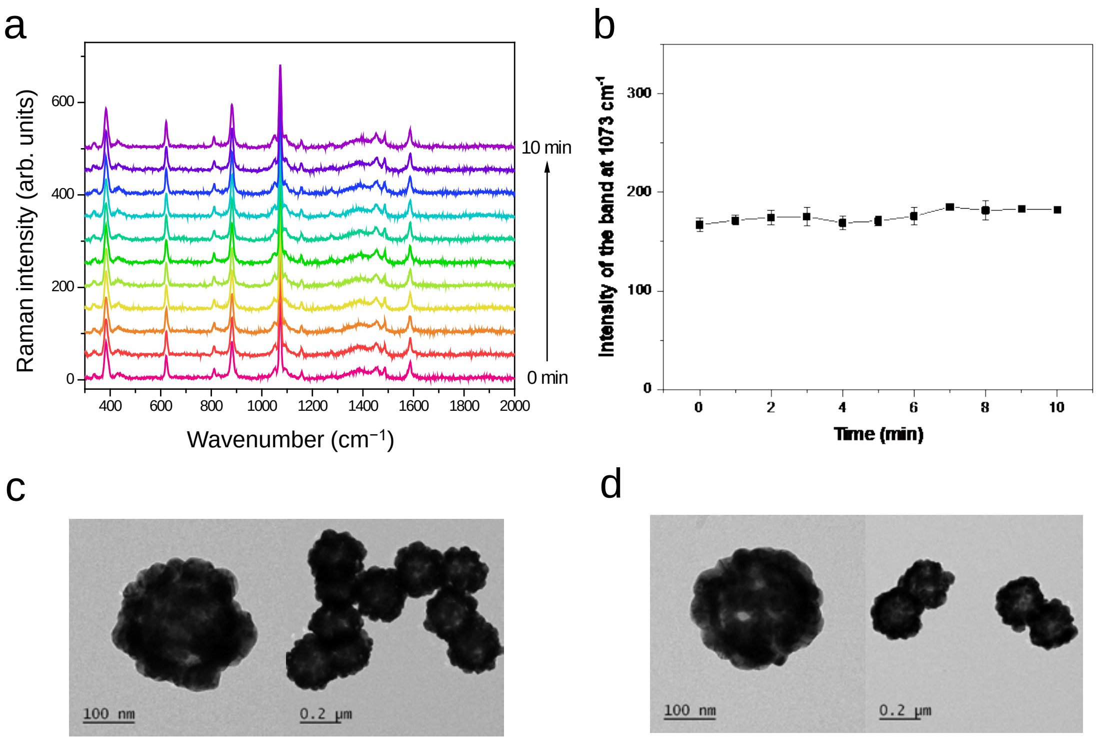

Figure S8. Photostability of Ag NGS particles. (a) Raman spectra of Ag NGS colloidal solution with continuous laser exposure for 10 minutes. The colloidal concentration of Ag NGSs was 0.1 mg/mL, and the Raman signals were collected from the sample in a capillary using 10× objective lens. Each spectrum was obtained with a 785-nm photo-excitation laser power of 6.53 mW and an 8-s acquisition time. (b) Signal intensity changes at 1073 cm^{-1} band during the Raman measurement in (a). TEM images of Ag NGSs (c) before the laser exposure and (d) after 10-minutes laser exposure.

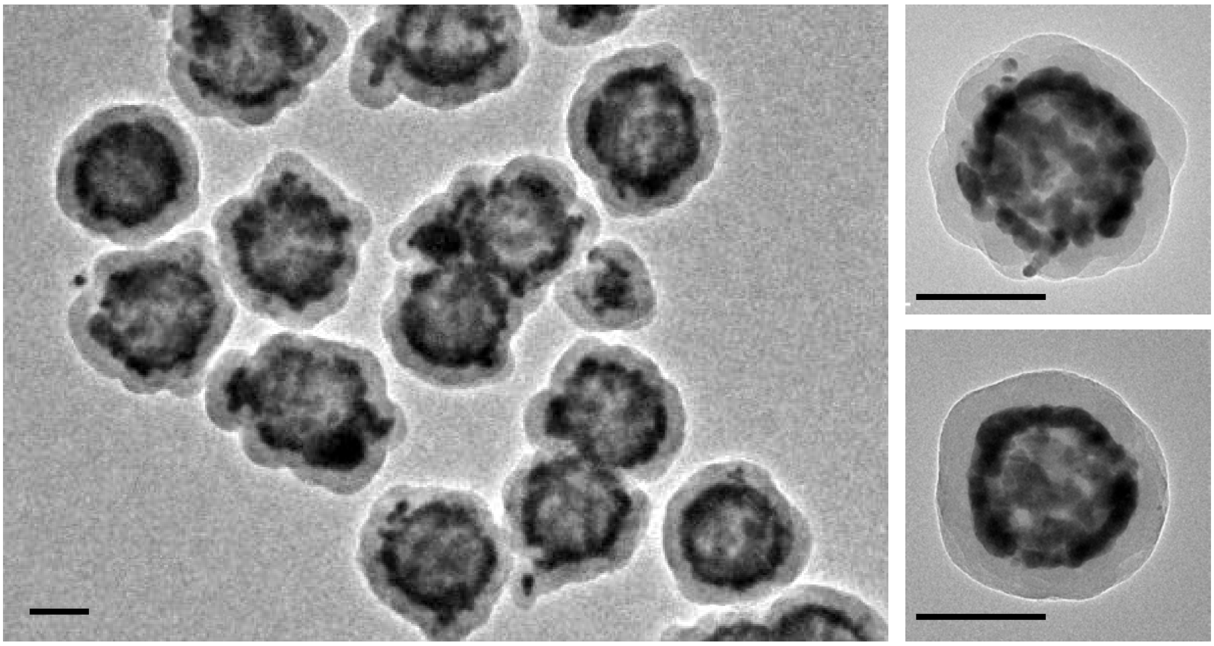

Figure S9. TEM images of silica-encapsulated Ag NGSs. Each scale bar represents 100 nm.

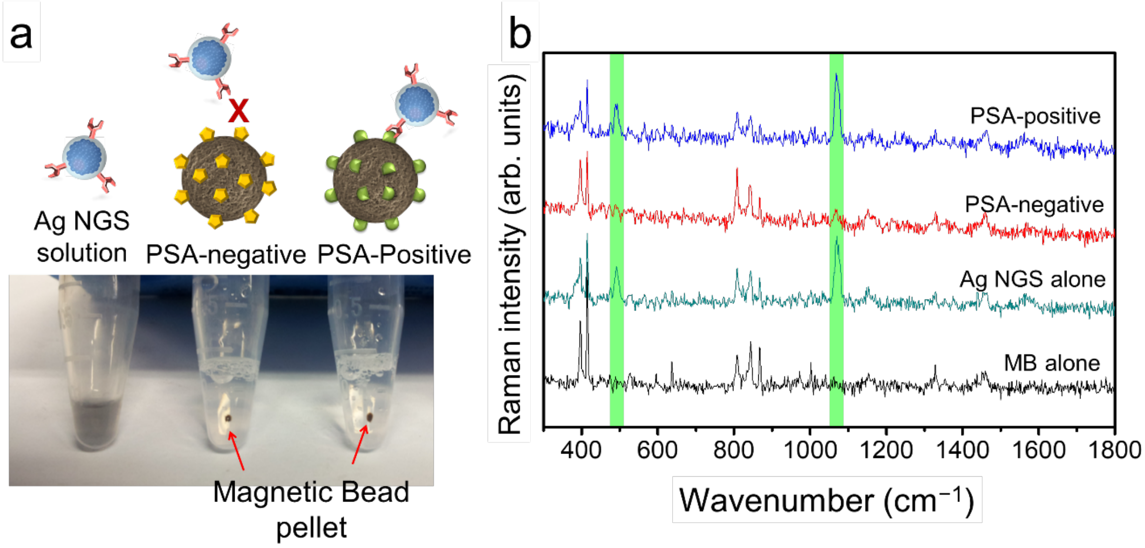

Figure S10. Conjugating PSA to epoxy-functionalized magnetic beads (MBs) to validate the Tab-AgNGS@SiO_2 probes. a) Photographs of antibody-conjugated Ag NGSs (probe solution), BSA-coated MBs in solution (a negative control), and a PSA-coated MB solution (positive sample). b) SERS spectra of a positive sample, a negative-control sample, the probe solution, and MBs alone (background)

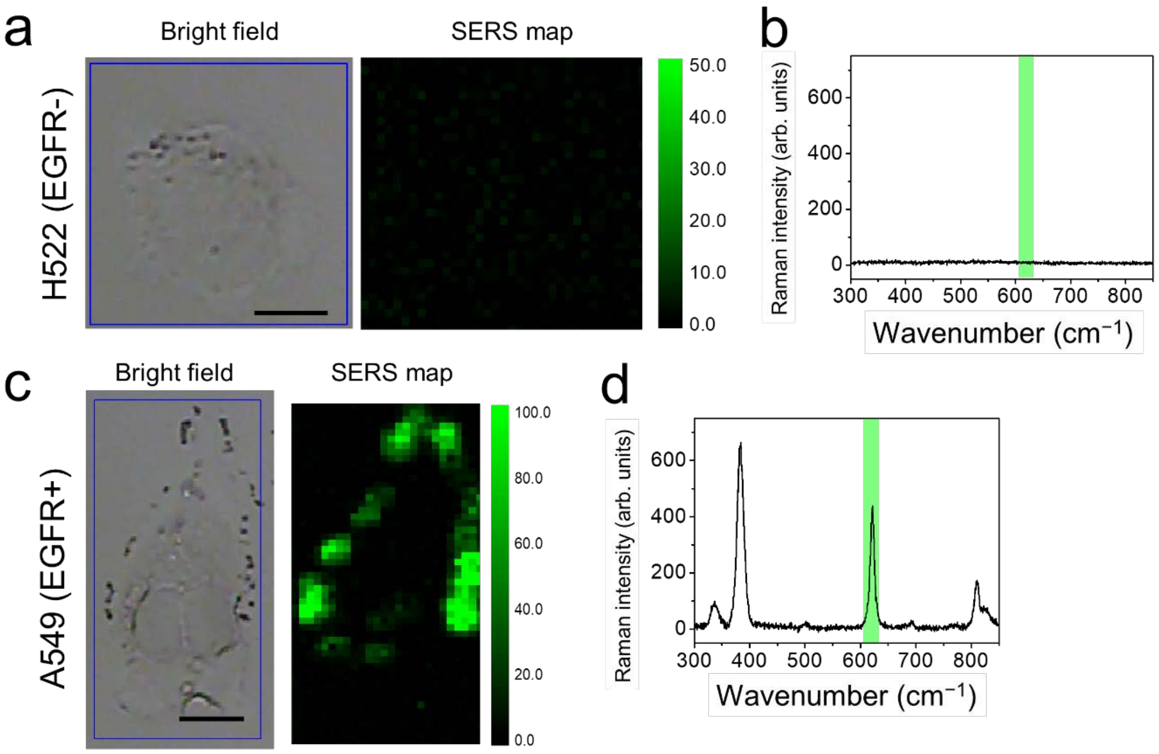

Figure S11. Bright-field images and corresponding SERS-mapping images for two different types of lung cancer cell lines. (a) A549 and (c) H522 lung cancer cells were treated with EGFR-Ag NGSs$_{[4\text{-}FBT]}$ particles. SERS maps were constructed using the 623 cm^{-1} band of 4-FBT SERS spectra with the (b) A549 and (d) H522 cell lines, using an excitation wavelength or 785 nm, a laser power of 7.5 mW, and a light-acquisition time of 1 s.
